# Using the electronic medical record to increase testing for HIV and hepatitis C virus in an Appalachian emergency department

**DOI:** 10.1186/s12913-021-06482-5

**Published:** 2021-05-29

**Authors:** Carmen N. Burrell, Melinda J. Sharon, Stephen Davis, Judith Feinberg, Elena M. Wojcik, Julia Nist, Owen Lander, Valerie Boley, Justin Burns, Ian B. K. Martin

**Affiliations:** 1 Department of Emergency Medicine, West Virginia University School of Medicine, 1 Medical Center Drive, Morgantown, West Virginia 26506 USA; 2 Department of Family Medicine, West Virginia University School of Medicine, Morgantown, West Virginia USA; 3 Department Of Health Policy, Management, and Leadership, West Virginia University School of Public Health, Morgantown, West Virginia USA; 4 Department of Behavioral Medicine and Psychiatry, West Virginia University School of Medicine, Morgantown, West Virginia USA; 5 Department of Medicine, Division of Infectious Diseases, West Virginia University School of Medicine, Morgantown, West Virginia USA; 6 West Virginia University Medicine, Information Technology, Morgantown, West Virginia USA; 7 West Virginia University Medicine, Emergency Service, JW Ruby Memorial Hospital, Morgantown, West Virginia USA; 8 Department of Emergency Medicine, Medical College of Wisconsin, Milwaukee, WI USA

**Keywords:** Human immunodeficiency virus, Hepatitis C virus, Electronic medical record, Best practice alert

## Abstract

**Background:**

The ongoing Appalachian opioid epidemic has led to increasing hepatitis C virus (HCV) infections among people who inject drugs (PWID), and Human Immunodeficiency Virus (HIV) outbreaks have been observed. The primary aim of this study was to assess the potential increase in screening for HIV and HCV in an academic central Appalachian emergency department (ED) through the use of Best Practice Alerts (BPAs) in the electronic medical record (EMR). A secondary aim was to assess for an increase in linkage to care using patient navigators.

**Methods:**

EMR algorithms based on current Centers for Disease Control and Prevention HIV and HCV testing recommendations were created that triggered Best Practice Alerts (BPAs), giving providers a one-click acceptance option to order HIV and/or HCV testing. Placards were placed in care areas, informing patients of the availability of routine screening. Patient navigators facilitated linkage to care for seropositive patients.

**Results:**

The BPA appeared 58,936 times on 21,098 patients eligible for HIV screening and 24,319 times on 11,989 patients eligible for HCV screening over a one-year period. Of those, 7106 (33.7%) patients were screened for HIV and 3496 (29.2%) patients were screened for HCV, for an overall testing increase of 2269% and 1065% for HIV and HCV, respectively. Linkage to care increased by 15% for HIV to 100, and 14% for HCV to 64%.

**Conclusion:**

HIV and HCV screening and linkage to care were increased in an academic ED setting in central Appalachia using EMR alerts. This approach could be utilized in multiple ambulatory settings. Increased testing and earlier linkage to care may help combat the current injection drug use-related HCV epidemic and avoid additional HIV outbreaks.

## Background

From 2003 to 2010, 3.5 million persons (range 2.5–4.7 million) were estimated to be infected with hepatitis C virus (HCV) in the United States [1]. HCV is a leading cause of advanced liver disease, and treatment of HCV-related diseases, including cirrhosis and hepatocellular carcinoma, is estimated to cost $6.5 billion per year [2, 3]. Injection drug use (IDU) is the primary driver of HCV infection and accounts for 60 to 70% of incident cases in the U.S. and other countries [4, 5]. The Appalachian region is currently in the midst of an injection opioid epidemic that is directly correlated with an HCV syndemic. Between 2006 and 2012, central Appalachia (Kentucky, Tennessee, Virginia and West Virginia) observed a 364% increase in acute HCV cases [6]. During this same time period, admissions to treatment for opioid use disorder in these states increased from 8.6 to 12% [6]. IDU is also a risk factor for HIV acquisition, and recently, HIV outbreaks among people who inject drugs (PWID) in West Virginia have been identified in Huntington and Charleston [7, 8]. In 2020, the CDC again updated the HCV screening guidelines to include a once in a lifetime screening for all adults 18 years of age and older except where the prevalence of the disease is < 0.1%, with additional testing for anyone with known risk factors [9].

### Importance

It is estimated that 45–85% of individuals are unaware of their HCV seropositivity [5]. Additionally, HCV co-infection with HIV has been observed at rates greater than 90% in HIV positive persons who inject drugs (PWID) [10]. The Centers for Disease Control and Prevention (CDC) has recommended HCV risk-based (e.g., injection drug use) screening since 1998, and in 2012 added a one-time HCV test recommendation for individuals born between 1945 and 1965, generally referred to as the “baby boomer” cohort, based on an observed 3.25% HCV prevalence in this cohort [11].

Traditionally, screening for infectious diseases in the emergency department (ED) has been driven by presenting complaint (e.g., fever, occupational bloodborne exposures, etc.) and clinical suspicion. Research has shown that this approach consistently misses cases, which supports the need for regular, non-clinically driven screening [12]. However, routine opt-out screening for HIV and HCV in this setting is often challenged by provider concerns over screening time, the time needed to link patients with positive results to care, and legal issues related to screening [13]. Consequently, physician-initiated testing has resulted in screening 1% of all patients presenting to the ED for HIV [13].

### Goals of this investigation

Gilead Sciences, Inc. established the Frontlines of Communities in the United States (FOCUS) program to promote routine, scaled-up screening (antibody plus confirmatory Ribonucleic Acid [RNA]) for HIV and HCV in the clinical setting [14]. A central component of the FOCUS program is the use of the electronic medical record (EMR) to assist in scaled-up testing. The FOCUS program TEST model contains four principles for routine screening: 1) testing integrated into normal clinical flow; 2) electronic medical record modification to support screening; 3) systemic policy change; and, 4) training, feedback, and quality improvement [14]. Previous studies have successfully used the EMR to increase screening for HIV and other infectious diseases [15–20]. However, all were conducted in urban areas not currently in the midst of a burgeoning opioid epidemic like the current one affecting central Appalachia.

We conducted a study based on the TEST model with support from Gilead Sciences, Inc., to assess for increased screening practices of HIV and HCV in an academic central Appalachian emergency department (ED) through the use of Best Practice Alerts (BPAs), a clinical decision tool triggered during patient visits for those eligible to receive only HIV screening, only HCV screening, or screening for both HIV and HCV according to CDC criteria. As a secondary aim, we assessed the impact of using patient navigators on linkage to care percentages.

## Methods

### Study design and setting

To assess our primary and secondary aims of accessing for increased number of HIV and HCV screenings in our emergency department and linkage to care of positive cases after implementing the TEST model, we conducted a pre-post study that used a historical control. Post-program implementation screenings ordered from June 2017 through the end of May 2018 were compared to the historical number of screenings ordered from July 2015 to July 2016. Linkage to care percentages for positive cases were also compared between these two periods.

Through the TEST model, an EMR-based protocol was implemented for routine HIV and HCV screening in a Level I trauma, tertiary care, academic medical center’s ED located in West Virginia. This ED has approximately 50,000 visits per year, with an average door to disposition time of 3.0 h for discharged patients. HIV screening was based on CDC guidelines to test individuals between ages 13 to 64 years at least once and high-risk individuals at least yearly [21]. However, due to the requirement of parental consent for pediatric patients, the site protocol was adjusted for testing to be performed on patients 18 to 94 years of age, both eliminating the need for patient consent for minors and to capture adults who may have not been previously tested. HCV testing was based on CDC guidelines at the time, testing all individuals born from 1945 through 1965, those with a recognized exposure, or those who are recognized as high risk [22]. FOCUS-supported studies define linkage to care as a first appointment with a provider within 3 months of testing.

### The electronic medical record

**Fig. 1:**
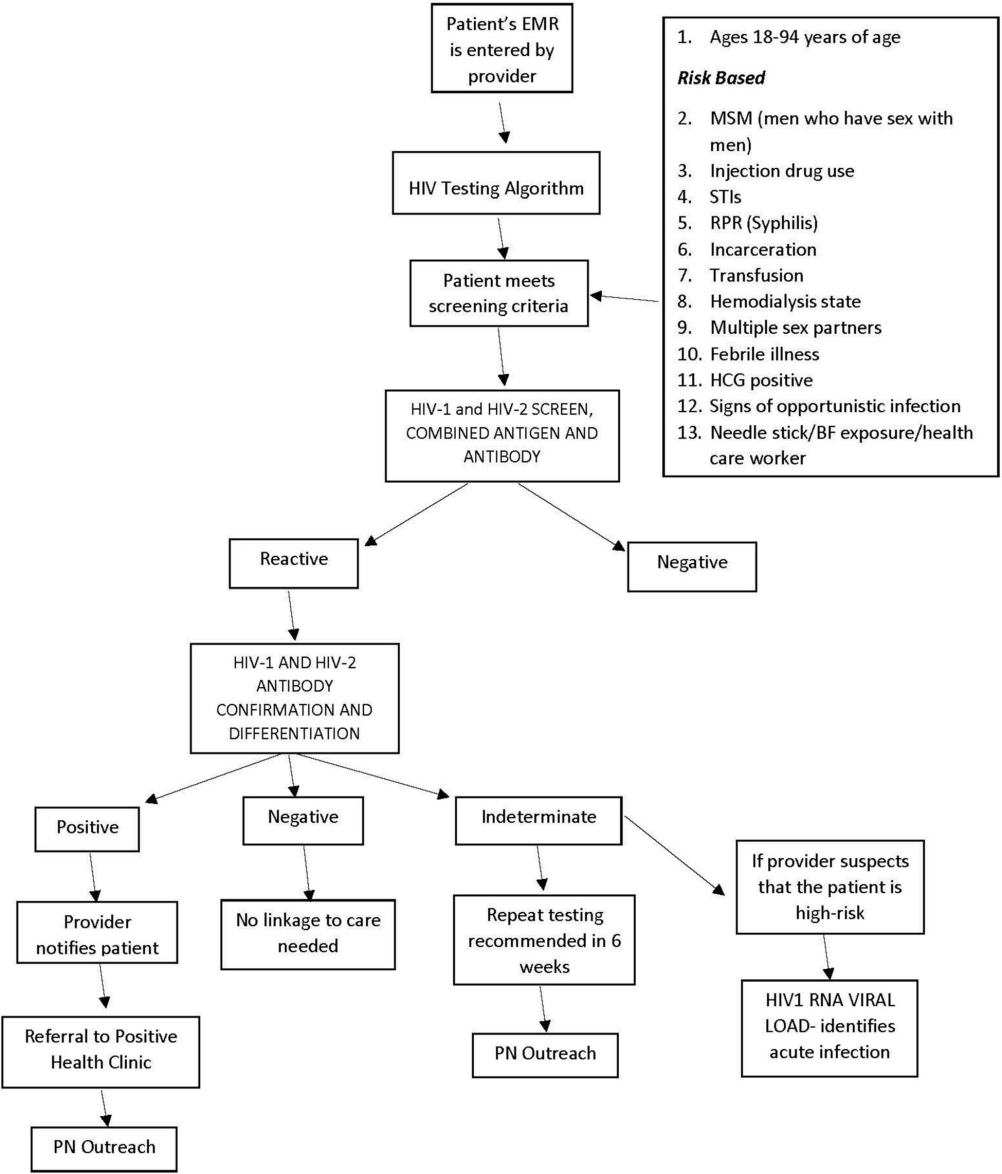
HIV screening and linkage to treatment testing algorithm utilized

**Fig. 2:**
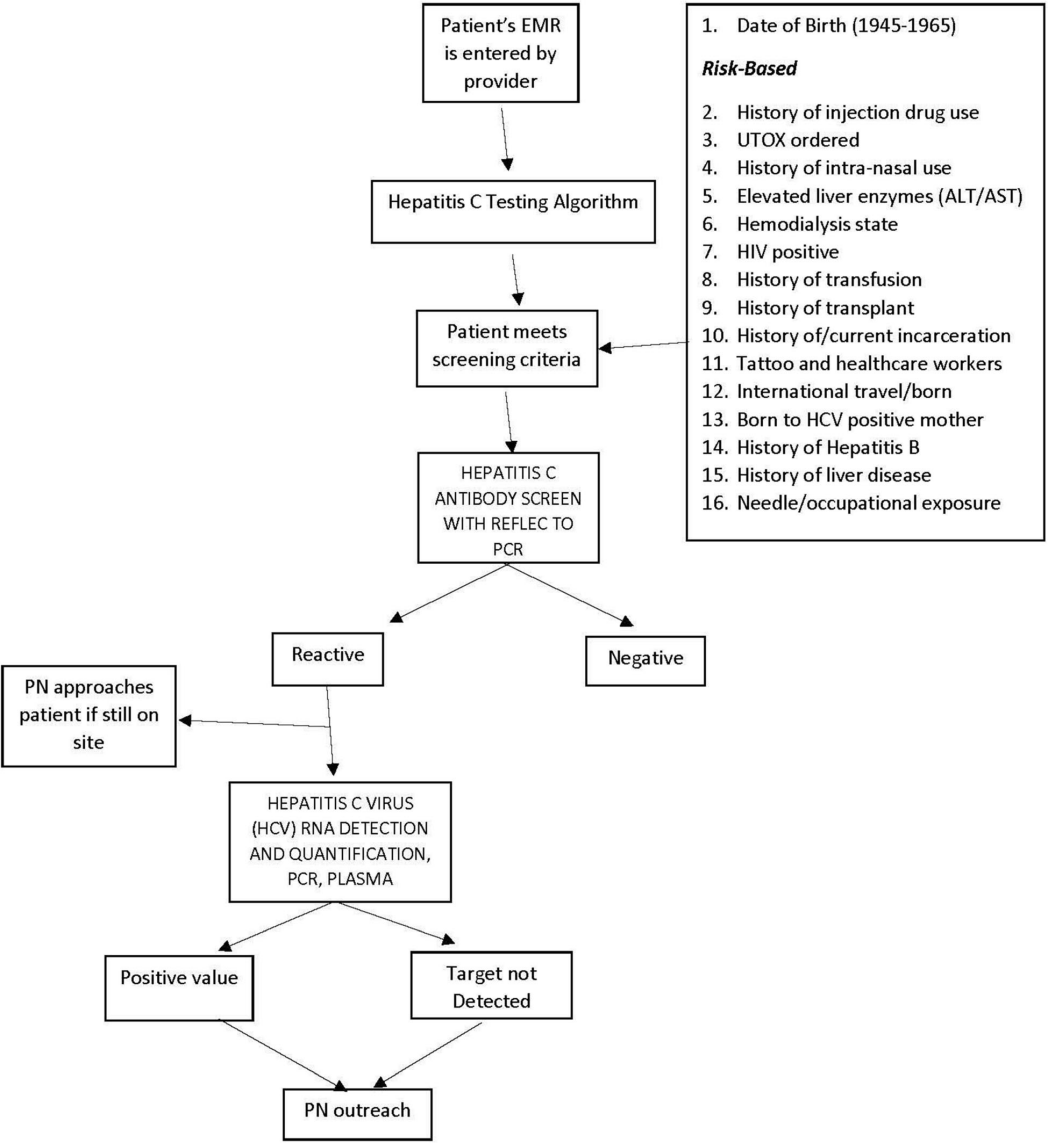
HCV screening and linkage to treatment testing algorithm utilized

To promote increased HIV and HCV screening within the TEST model, we designed BPAs in our EMR (Epic® 2015, Epic Systems Corporation) based on CDC guidelines for HIV (Fig. 1) and HCV testing (Fig. 2) adjusted for age as described above. If the EMR found the patients eligible based on age cohort and/or risk factors, the BPA would be triggered and appear to providers and staff upon opening the orders tab. The risk factors were reviewed from both the patient’s problem list and past medical history. The BPA would trigger for low-risk patients every 12 months to access risk and need for testing if not previously performed and quarterly for any high-risk patients with risk factors. Individuals without risk factors were not removed from this yearly evaluation as it prompted providers to reassess the need for screening in case there were new additions to the patient history. After approval by the hospital clinical decision support team responsible for all BPA requests, an Epic® ED module analyst expended roughly 20 h of time building and testing the BPA algorithm. Upon presentation of the BPA, providers and nursing staff could select from the following options if they decided to not order screening: “Will Assess,” “Not Clinically Appropriate,” and “Patient Refused.” [23] When providers chose “Will Assess,” the BPA would continue to appear until orders were placed or another option was chosen; however, a hard stop was not employed and charts could be closed without an acceptance or rejection of the order.

### Provider training

Attending and resident physicians, advanced practice providers (APPs), and nursing staff were instructed on both the BPAs and the screening eligibility criteria to prepare for implementation. A live training session was conducted with all providers at a regularly scheduled staff meeting, per the TEST principle that encourages training, feedback and quality improvement. Thereafter, brief monthly program updates were provided at these meetings, allowing for opportunities for clinician feedback. Additionally, frequent electronic reminders with protocol details and BPA screenshots were sent from physician and nursing leadership to all providers and staff. These trainings and educational efforts were vital, given the fact that few alerts appeared within the EMR environment prior to this initiative. Preventative screening BPAs occur less frequently in the ED than in primary care where BPA fatigue can influence provider responsiveness.

### Implementation of screenings

The BPA-triggered HIV and HCV screening was implemented in June 2017. To facilitate testing, the BPA was configured, by risk factors and age, to trigger at multiple points in the patient care process, such as during nursing triage and provider evaluation. The BPA required only one “mouse click” for the provider to order each screening test. Placards were hung in all treatment and triage areas to inform patients. The provider or nursing staff would also inform the patient of any planned screenings, and, in turn, the patient would have the opportunity to opt out of screening for HIV and/or HCV at time of evaluation (Figs. 1 and 2). If a patient did not express the desire to opt out, HIV/HCV testing was conducted on the same blood drawn for routine initial tests if the patient met the screening criteria. This procedure obviated the need to draw an additional serum sample later during the visit. The patient would also have the opportunity for testing if no laboratory testing work was planned, and the provider would have the opportunity to defer testing if not clinically appropriate or the patient refused. If a previous diagnosis of HIV or HCV was not appropriately documented within the past medical history, the BPA would still be triggered for screening.

### Laboratory testing

The HIV screening test utilized by the hospital laboratory is a fourth-generation combined antigen and antibody chemiluminescent immunoassay test that reflexes automatically to an antibody differentiation immunoassay for confirmation. Nucleic acid testing is performed at the provider’s discretion for patients deemed at higher risk for acute infection (Fig. 1). The HCV screening test utilized is a chemiluminescent microparticle immunoassay performed on the ARCHITECTi® platform that reflexes automatically to quantitative HCV RNA testing, if the initial antibody test result is positive (Fig. 2). HIV confirmatory results were available for viewing within twelve hours, and HCV RNA results were available within 3 days although initial antibody tests could be available within a few hours. All tests are completed in real time. All HIV and HCV antibody and confirmatory tests were free of charge through grant funding from the FOCUS program.

### Patient navigators and linkage to care

Upon a positive screening result, a member of the patient’s care team initially notified the patient of his/her antibody results if the patient was still in the ED, and when available, patient navigators (PNs) would also meet with the patient in the ED to assist with linkage to care.

Patients that screened positive and were dispositioned elsewhere were initially contacted by the PNs. PNs built rapport with patients and informed them that all their questions would be answered at the time of their appointment with the referred specialty provider. PNs were trained by clinical faculty and staff of the Department of Emergency Medicine at the West Virginia University School of Medicine and were also Health Insurance Portability and Accountability Act (HIPPA) compliant. PNs were responsible for linkage to care only for FOCUS program participants.

Follow-up appointments were scheduled with infectious diseases (ID), behavioral medicine, digestive diseases, gastroenterology, or primary care, which were all considered appropriate linkage to care. PNs called patients to discuss patient awareness of test result(s) and available appointment options. If a patient screened antibody positive for HIV or HCV during daytime hours, the PNs met with the patients in the ED to inform them of their antibody results and that someone would be in touch via telephone to give confirmatory results and provide linkage to care in the next few days. PNs worked closely with schedulers in each department in order to expedite follow-up appointments or re-testing when necessary. When following up with patients after initial contact, PNs gave patients the opportunity to choose texting as the main form of contact, as opposed to future phone calls. This was the first FOCUS program to offer a texting option for the primary form of communication.

Patients who screened HIV-positive and had a subsequent positive confirmatory result were referred to the West Virginia University Positive Health Clinic for immediate follow-up and further evaluation. The Positive Health Clinic, supported in part by the Ryan White Care Act, provides state-of-the-art, comprehensive HIV care services to a largely rural, medically underserved area where access to HIV care is very limited. Patients who initially screened positive for HIV but had a negative or indeterminate confirmatory result were contacted by the PNs and encouraged to have repeat HIV testing in 6 weeks, due to the potential risk of early infection. When possible, these patients were scheduled to return to one of our primary care or urgent care locations for testing, instead of an additional ED visit. For patients who were identified HIV positive but were not new infections, the PNs ensured that they were still linked to care in a follow-up clinic.

Patients were contacted by PNs and subsequently referred for HCV follow-up appointments with infectious disease (ID), digestive diseases, behavioral medicine, or a primary care clinic upon an initial positive antibody screening result, regardless of confirmatory testing status. This was due to the risk of a previously active infection or potential of having risk factors requiring follow-up. Most patients were seen by ID; however, other clinic referrals, such as digestive diseases clinic, were made based on patient preference or could be deferred if the patient had previously scheduled primary care or behavioral medicine appointments. All patients who had previously scheduled appointments with primary care or behavioral medicine were also contacted by PNs so that HCV status would be known. Patients were asked to inform the provider of their status at the time of their scheduled appointment so that the provider could refer the patient to follow-up care.

Patients who were not successfully linked to care within 90 days were considered to be “lost to follow-up” and were not contacted again by PNs. Patients could be linked to care after 90 days if they reached out to PNs via call or text to the PN. However, there are a small percentage of patients who are considered to be “lost to follow-up” before the 90-day period; reasons for this included incarceration, death, and refusing an appointment. Although PNs stressed the importance of attending an appointment, patients who were uninterested were asked to reach out to the PN if a different decision was made after consideration.

In order to enhance patient follow-up, PNs offered transportation assistance to patients to decrease that barrier to linkage to care. PNs could coordinate taxi transport with patients who did not have their own means of transportation, offer gas gift cards to those who had a reliable source of transportation but needed help affording the trip or provide information on local bus transit routes close to their residence. In addition, PNs also offered to accompany patients to their first appointment.

PNs only provided linkage to care and follow-up to newly identified cases through the FOCUS program. Often, previously diagnosed patients would not identify and would be tested through the screening process. When this occurred, the patients were contacted and linked to follow-up care if not previously established.

### Data analysis

The following data points were extracted from the EMR to assess our primary aim: number of BPA appearances per patient for HIV and HCV, number of HIV and HCV screening orders and patients tested, positive screening and confirmatory results for HIV, antibody-positive and RNA-positive results for HCV. We tracked the number of patients successfully linked to care within 90 days to assess our secondary aim. Demographics (age, gender, race) and risk factors (i.e., history of IDU) were obtained based on real-time chart reviews for each patient with a positive screening test and results from the EMR were entered into a log by the PNs. All data points were analyzed descriptively to provide percentages and appearance rates for the BPA and linkage to care percentages.

## Results

**Fig. 3:**
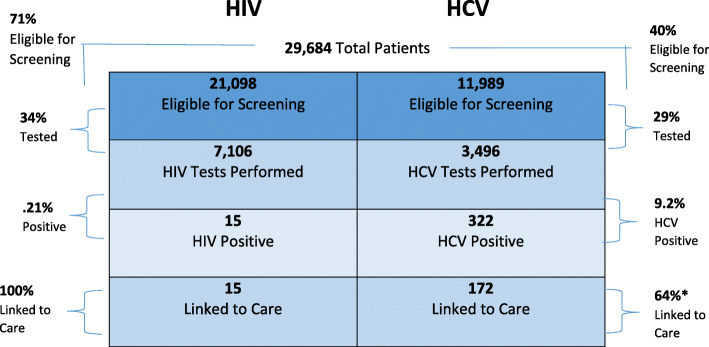
HIV and HCV screening statistics and linkage to treatment rates

Prior to implementation, approximately 300 HIV screenings and 300 HCV screenings were conducted in the ED between July 2015 and July 2016, and only 85 and 50% respectively of those with positive results were linked to care as a result of testing. From June 5, 2017 to May 31, 2018, 29,684 adult patients presented to the ED. The BPA appeared 58,936 times on 21,098 patients eligible for HIV screening, for a rate of 2.8 times per patient. The BPA appeared approximately 24,319 times on 11,989 patients eligible for HCV screening based on risk factors or lack of prior screening, for a rate of 2.0 times per patient. Of those eligible, 7106 (34%) patients agreed to be screened for HIV and 3496 (29%) patients for HCV (Fig. 3), which is a 2269% and 1065% increase in HIV and HCV screening, respectively, in 12 months.

Of the 322 HCV positive patients, 54 were excluded from the linkage to treatment rate for the following reasons: 35 patients were scheduled for an appointment further in the future, 15 patients were incarcerated, and four patients were deceased.

Twenty-eight patients screened HIV-positive and 15 (53.6%) had positive confirmatory results; of those, only one was newly diagnosed. The average age of HIV-positive patients was 39 years (range 19 to 62 years); the majority were male (60%), white (60%), and non-Hispanic (80%). According to chart review, three of 15 (20%) stated their method of acquisition was related to IDU (Table 1), and all were linked to care through the Positive Health Clinic (100%). The remaining 13 patients had negative or indeterminate HIV confirmatory results; all were scheduled for additional testing and were subsequently determined to not have HIV infection.

**Table 1:** HIV and HCV-positive patient demographics

	HIV N (%) Total *N* = 28	HCV N (%) Total *N* = 322
**Sex**		
**Male**	17 (60)	183 (57)
**Female**	11 (40)	139 (43)
**Age**		
**Range**	19–62	20–76
**Average**	39	43
**Race**		
**White**	17 (60)	290 (90)
**Other**	11 (40)	32 (10)
**Ethnicity**		
**Non-Hispanic**	23 (80)	312 (97)
**Hispanic**	5 (20)	10 (3)
**IDU**		
**Yes**	3 (20)	235 (73)
**VNo**	25 (80)	87 (27)
**“Baby Boomer”**		
**Yes**	N/A	117 (36)
**No**	N/A	205 (64)
**MSM**		
**Yes**	7 (25)	N/A
**No**	21 (75)	N/A
**Heterosexual** **Contact**		
**Yes**	6 (21)	N/A
**No**	22 (79)	N/A
**Confirmed Positive**		
**Yes**	15 (54)	191 (59)
**No**	13 (46)	131 (41)

*IDU Injection drug user; *Baby Boomer* Born between 1945 and 1965, *MSM* Men who have sex with men

Overall, 322 patients screened antibody-positive for HCV, with 191 (59%) having a positive RNA confirmatory result; of the confirmed positive results, 141 (74%) were newly identified. The average age of HCV antibody-positive patients was outside the risk factor group of the “baby boomer” cohort at 43 years (range 20 to 76 years); the majority were male (57%), white (90%), and non-Hispanic (97%). Following extensive chart review, 73% were found to have a history of IDU (Table 1). All 322 patients with antibody-positive HCV results were referred to the infectious diseases and/or digestive diseases clinics for follow-up. The PNs were able to link 172 of 268 eligible patients (64%) to their first follow-up appointment within 3 months, with 35 (13%) having a future scheduled appointment, 15 (6%) incarcerated, and four (1%) deceased (Fig. 3). Five patients were co-infected with HIV and HCV. Although only seven gas gift cards were provided to facilitate linkage to care, all seven patients successfully attended their first appointment.

## Discussion

This study is the first to highlight the challenges and successes of implementing an HIV and HCV EMR-based screening in an ED serving rural, central Appalachia using the TEST model. Using an EMR to prompt providers to order HIV and HCV screening based on CDC guidelines that were age-adjusted was highly successful in increasing screening rates for both infections; it also showed that testing outside of the ages recommended by CDC guidelines established a high number of positives. An increase in linkage to care rates for both HIV and HCV seropositive individuals with the use of PNs was observed, and this was the first FOCUS program to successfully utilize texting as the primary mode of communication to assist in linkage to care. Previous studies have implemented similar EMR-based infectious diseases screenings in the ED; however, these studies were conducted entirely within urban areas. When compared to our urban ED counterparts, our patient population screening antibody-positive for HCV was slightly lower in age on average than the established “baby boomer” cohort for whom testing is recommended [3]. By using EMR-programmed, reflex testing for antibody-positive patients in the hospital laboratory, a 100% HCV RNA confirmatory testing rate was achieved. This is significantly higher than the 40–50% HCV RNA testing rate described in the literature [15]. Furthermore, the BPA required fewer “mouse clicks” (two total) for the provider to order both screening tests than prior studies [20]. The ease of ordering an HIV and/or HCV test in the ED may have resulted in an increased order rate. Configuring the BPA to trigger at multiple points in the patient care process (i.e., triage, in-room, etc.) promoted increased screening rates. In addition, by setting the BPA to trigger at regular intervals based on patient risk, the provider was reminded to access any changes in patient status and the need for the screening labs if not previously performed or if risk factors had changed.

Most impressively, the linkage to care rates of 64% (HCV) and 100% (HIV) are among some of the highest reported, which has typically been shown to vary between 30 and 40% for HCV and 60–80% for HIV seropositive patients, respectively [17, 18, 20]. Not only were newly identified infections linked for follow-up care but chronic infections who may have been lost to follow-up were assisted with linkage to care. These rates may be higher than prior studies for a number of reasons.

First, PNs were able to contact patients via texting per patient preference, and texting as a means of contacting patients—particularly younger patients—has been suggested as a successful strategy for hard-to-reach patients such as PWID and is novel to our FOCUS study [24]. A recent study among PWID in San Diego observed that the majority used mobile technology for voice, text and/or internet access, with high mobile technology use associated with younger age [24]. Other studies have noted that accompanying patients to their appointment increases the patient’s likelihood of attending [15].

Second, the PNs worked closely with the schedulers within multiple specialty clinics to ensure patients were scheduled within 2–4 weeks of their original ED visit, well within the FOCUS program requirement for a first visit to be scheduled within 3 months. PNs contacted patients soon after their ED visit to convey confirmatory results and to schedule referral to a specialist shortly thereafter. Third, since transportation can be a significant problem in rural areas, providing transportation assistance promoted attendance at follow-up appointments. Lastly, as our ED is part of a large healthcare system in central Appalachia, a breadth of follow-up referral options was available for successful linkage to care. Keeping patients connected through the extensive healthcare system likely increased the chances of linkage to care, as has been noted by similar studies [15].

### Implementation challenges

Success notwithstanding, a few notable challenges were encountered. First, during the initial 2 weeks of implementation, the BPA was scheduled to appear upon the provider and nursing staff entering the patient’s chart at any time. This design led to an increased rate of inappropriate BPA appearances. More specifically, providers relayed that they did not have sufficient information about patients to address the BPA upon initially entering the patient’s chart at the beginning of the patient visit. The BPA would continue to trigger until an option was chosen and the chart was closed; the BPA would also inappropriately trigger during order encounters or addendum to patient visits. As a result of this feedback, the location of the BPA was moved to occur upon opening the orders tab of the patient’s chart; this was the preferred location by the providers and proved to be the most logical option during the course of care. If no orders were placed, the BPA would not be triggered.

Second, the EMR was programmed to appear based on specified risk factors as outlined by the CDC and modified based on age [21–23]. For the BPA to trigger, specified risk factors must be populated in searchable areas of the EMR— for example, the problem list or past medical history tabs. However, it was discovered during the study that these tabs— and other searchable areas of the EMR— are often not populated with the most up to date patient information. Similar findings regarding risk-based screenings via the EMR have been reported [21]. Often, these questions concerning risk factors may be addressed but only added to the body of the patient note. One study found that over 80% of participants reported having ever avoided telling a clinician medically relevant information [25]. Failure to disclose information can decrease patient care, and even lead to patient harm [25]. Therefore, future strategies to update this risk information, as well as the triage complaint, captured in the EMR during the patient visit are being explored to maximize the identification of patients for whom CDC recommended screening is indicated.

Another aspect of the BPA’s initial programming was for the BPA to appear upon certain chief complaints assigned to the patient during nursing staff triage procedures. Initially, the BPA would not be triggered for the physician or APP if the nursing staff had dismissed the alert. Based upon provider feedback, the algorithm was modified to trigger the BPA in the orders section of a provider chart even if a nurse initially selected “Not Clinically Appropriate” or “Patient Refused” at triage. This modification gave providers the option of assessing whether or not testing was indicated by further clinical information gathered during the course of evaluation and provided additional patient education opportunities regarding screening benefits.

Although our PNs were some of the most successful in the FOCUS program to link patients to care, this success also came with its challenges. PNs frequently could not connect with patients on the first attempt due to a lack of contact information available in the patient’s chart. If patients did not provide sufficient or accurate contact information upon presentation to the ED, the PNs could not follow-up. Future studies should examine the addition of registration personnel to the navigation team to ensure that patients are providing multiple, accurate points of contact, so that PNs can ensure successful follow-up and linkage to care.

### Future directions

The process of deploying the HIV/HCV testing BPA in additional hospital EDs that have recently adopted Epic® should be feasible in the future. Also, the process of implementing universal screening for HCV driven by age criteria to address the difficulties encountered with configuring the BPA to trigger based on unavailable risk factors should be explored. Finally, the testing rates by provider should be examined to investigate ordering discrepancies to further maximize the screening rates. As this screening process becomes integrated into the care model, we hope that future guidelines will broaden the categories of risk factors and insurance will cover these screening tests to a greater extent.

## Limitations

Our study is based on pre-post implementation at a single site. Thus, in addition to the limited generalizability of our findings to other sites, our results are subject to confounding. Future studies could provide some control for this confounding by including a separate comparison site. Second, the largest limitation is the omission of documented critical information in the EMR. While the goal was to follow CDC testing guidelines for patient risk factors, these risk factors are not always present in a readily accessible format in the EMR. This results in a gap in which eligible patients go untested, and their HIV and/or HCV status remains unknown. While the EMR was able to query data from the patient’s problem list, past medical history, and diagnosis at time of visit to identify eligibility for testing, this information is often missing or inaccurate, or it may be documented in other segments of the medical record such as the history narrative. We hoped that by adjusting screening criteria based on age wider than the CDC guidelines, we would capture this missing population. Risk factors frequently remain unknown if not discussed at the time of visit or if the patient fails to disclose key information such as IDU. Lastly, the PNs frequently encountered the obstacle of incorrect or missing patient contact information and therefore could not link patients to care or further follow-up testing when needed.

## Conclusion

Introducing an EMR based HIV and HCV screening program based on CDC screening guidelines modified by age into an academic, Appalachian ED using the TEST model increased the number of HIV and HCV screenings and identification of positive cases. Using patient navigators also improved linkage to care percentages for identified positive cases. This model may help identify persons with HCV for curative therapy, while helping prevent an HIV outbreak due to the substantial regional increase in IDU. Future studies should examine the impact of modifying EMRs to better capture risk-based information on testing rates pre- and post-implementation of an EMR-based intervention. Strategies to promote the capture of accurate contact information for the hard-to-reach PWID population and further improvements to linkage to care rates for patients testing positive for HIV and/or HCV are also needed.

## Data Availability

The datasets generated and/or analyzed during the current study are not publicly available due to IRB restrictions to protect sensitive information.

## Abbreviations

HCV: Hepatitis C Virus
HIV: Human Immunodeficiency Virus
EMR: Electronic Medical Record
BPA: Best Practice Alert
CDC: Centers for Disease Control and Prevention
IDU: Injection Drug User
PWID: People Who Inject Drugs
ED: Emergency Department
FOCUS: Frontlines of Communities in the United States
RNA: Ribonucleic Acid
APPs: Advanced Practice Providers
PNs: Patient Navigators
HIPAA: Health Insurance Portability and Accountability Act
ID: Infectious Disease
